# Measuring Access to Medicines: A Survey of Prices, Availability and Affordability in Shaanxi Province of China

**DOI:** 10.1371/journal.pone.0070836

**Published:** 2013-08-01

**Authors:** Minghuan Jiang, Shimin Yang, Kangkang Yan, Jun Liu, Jun Zhao, Yu Fang

**Affiliations:** 1 Department of Pharmacy Administration, School of Pharmacy, Health Science Centre, Xi'an Jiaotong University, Xi'an, China; 2 Department of Population Medicine, Harvard Medical School and Harvard Pilgrim Health Care Institute, Boston, Massachusetts, United States of America; UNAIDS, Switzerland

## Abstract

**Objective:**

To measure the prices and availability of selected medicines in Shaanxi Province after the implementation of new healthcare reform in 2009.

**Methods:**

Data on the prices and availability of 47 medicines were collected from 50 public and 36 private sector medicine outlets in six regions of Shaanxi Province, Western China using a standardized methodology developed by the World Health Organization and Health Action International from September to October 2010. Medicine prices were compared with international reference prices to obtain a median price ratio. Affordability was measured as the number of days’ wages required for the lowest-paid unskilled government worker to purchase standard treatments for common conditions.

**Findings:**

The mean availabilities of originator brands and lowest-priced generics were 8.9% and 26.5% in the public sector, and 18.1% and 43.6% in the private sector, respectively. The public sector procured generics and originator brands at median price ratios of 0.75 and 8.49, respectively, while patients paid 0.97 and 10.16. Final patient prices for lowest-priced generics and originator brands in the private sector were about 1.53 and 8.36 times their international retail prices, respectively. Public sector vendors applied high markups of 30.4% to generics, and 19.6% to originator brands. In the private sector, originator brands cost 390.7% more, on average, than their generic equivalents. Generic medicines were priced 17.3% higher in the private sector than the public sector. The lowest-paid government worker would need 0.1 day’s wages to purchase captopril for lowest-priced generics from private sector, while 6.6 days’ wages for losartan. For originator brands, the costs rise to 1.2 days’ wages for salbutamol inhaler and 15.6 days’ wages for omeprazole.

**Conclusions:**

The prices, availability and affordability of medicines in China should be improved to ensure equitable access to basic medical treatments, especially for the poor. This requires multi-faceted interventions, as well as the review and refocusing of policies, regulations and educational interventions.

## Introduction

Access to essential medicines is integral to fulfilling the rights of citizens to the highest standards of health. However, at least one third of the world’s population has no regular access to medicines [1]. Numerous reasons exist for the lack of access to essential medicines, but in many cases high drug prices are a major barrier.

In China, total expenditure on health care in 2010 was 4.92% of gross domestic product (GDP), just under the 5% level recommended by the World Health Organization (WHO). In 2010 Chinese national healthcare expenditures were USD 289.6 billion, with out-of-pocket health expenditure of nearly USD 102.8 billion. Pharmaceuticals account for about half of total health spending in China, representing 43.4 percent of spending per inpatient episode and 52.2 percent of spending per outpatient visit [2]. This proportion is one of the highest in the world, and compares to an average of around 17% in the OECD countries [3]. The high cost of medical care services and medicines is considered the major obstacle in accessing health care in China [4].

The new round of healthcare reform launched in 2009 included a number of measures to tackle high medical costs, such as increasing government spending to provide for all of those in need, establishing a national essential medicines system to reduce medicines prices, and promoting free medical treatments and advocating prevention. According to this healthcare reform plan, by 2012, all primary health care institutions (namely urban community health care centers, rural township hospitals, and village clinics) receiving government subsidies will be required to stock and dispense essential medicines at zero mark-up [5]. Shaanxi Province, with a population of nearly 37.62 million people, is located in Western China and has 11 cities with Xi'an as the capital. The Shaanxi Government initially piloted the essential medicine policy with zero mark-up in all primary health care institutions of two cities (Yulin and Baoji) in June 2009, and then implemented it in other cities after November 2010. Primary health care institutions thus can only stock and dispense medications currently included in the National Essential Medicine List (NEML) or Provincial Essential Medicine List (PEML). All medicines stocked by primary health care institutions are procured from the Department of Drug Price Bidding at the Shaanxi Provincial Department of Health under a system of “unified bidding, unified pricing, and unified distribution”, and sold with zero mark-up [6]. Meanwhile, the National Development and Reform Commission (NDRC) regularly lowers medicine prices to ease the burden of patients. In theory, these changes should reduce inefficiencies in drug delivery [7]. However, governments have little information on supply, pricing, distribution, and the use of drugs to help them construct sound medicine pricing policies or evaluate their impact [8].

In May 2003, the World Health Organization (WHO) collaborated with Health Action International (HAI) to develop a standardized method for surveying medicine prices, availability, affordability, and price components in low- and middle-income countries [9]. Three surveys have been carried out using the WHO/HAI methodology, in Shandong [10], Shanghai [11] and Hubei [12], and all revealed an alarming lack of access to affordable essential medicines in both the public and private sectors in China. These three surveys were conducted in eastern and central China from 2004 to 2008, prior to China’s new healthcare reform, and research on the availability and use of essential medicines in China’s underdeveloped western regions is scarce. The main goal of this study was to clarify medicine pricing in China by conducting a cross sectional survey to measure the prices, availability and affordability of a standardized set of medicines in Shaanxi Province, Western China from September to October 2010, utilizing the WHO/HAI methodology. To our knowledge, this is the first study of its type in China since the implementation of the essential medicine system in 2009. Previous work on this area has been limited to descriptive studies [13]–[15] and one survey [16] that analyzed the essential medicines list, policy trends and prescribing behaviors.

## Methods

### Ethics Statement

Xi'an Jiaotong University Health Science Center, Shaanxi Provincial Department of Health and Shaanxi Food and Drug Administration approved the study prior to data collection.

### Sampling

A systematic sampling method was used to select medicine outlets. The major urban centre of Xi'an was selected as one survey area, and an additional five areas reachable within one day’s drive from Xi'an were randomly chosen. The final sample comprised the following six survey areas: Xi'an, Yulin, Xi'anyang, Baoji, Shangluo and Weinan. In each survey area, the sample of public sector medicine outlets was identified by first selecting the main public hospital. An additional four public medicine outlets per survey area were then randomly selected from those within four hour's drive from the main hospital. The public sector sample therefore contained five public medicine outlets in each of the six survey areas, for a total of 30 public outlets. The private sector sample was identified by selecting the private sector medicine outlet closest to each of the selected public medicine outlets, yielding a total of 30 private outlets. Back-up facilities were selected in case the availability of the survey medicines was less than 50% at a given outlet. The sample of public and private medicine outlets is listed in Table S1.

### Selection of Medicines to be Surveyed

Among 47 medicines included in the survey, 27 belonged to the core list medicines suggested by WHO/HAI for international comparison, and 20 were supplementary drugs. The core list medicines were selected on the basis of the global disease burden. The supplementary medicines were selected based on local importance, the NEML and disease burden, and finalized after expert opinion and feedback from international experts (from HAI and WHO) and an advisory committee (including practicing pharmacists, academics, and experts from the Drug Administration Authority of Shaanxi Province). Table S2 lists all the survey medicines. Of the 47 medicines surveyed, 33 were on the NEML. Since our survey started prior to the issue of the PEML in October 2010, three medicines listed on the PEML subsequently were no longer considered essential medicines. For each medicine, information was collected on the availability and price of both the originator brand (OB), and the lowest-priced generic (LPG) equivalent found at each medicine outlet. The LPG was determined at the facility level.

### Data Collection and Entry

Twelve trained data collectors organized in pairs to visit medicine outlets and recorded medicine availability and price using a standardized form. Three prices were recorded, namely the procurement price and patient price in public medicine outlets, and the patient price in private pharmacies. Procurement data were generally obtained from the Shaanxi government procurement office [17], where procurement data were unavailable, procurement prices were collected at individual medicine outlets. Survey data were entered into the pre-programmed Excel Workbook (WHO/HAI 2008) by two people using a double entry technique. For a more detailed description of the survey method, see the Methods section of the manual published by WHO/HAI [18].

### Statistical Analysis

The availability of individual medicines is calculated as the percentage (%) of medicine outlets where the medicine was found. Mean availability is also reported for the overall “basket” of medicines surveyed. The availability data only refer to the day of data collection at each particular facility and may not reflect average monthly or yearly availability of medicines at individual facilities.

As an external benchmark, and to facilitate cross-country comparisons, medicine prices obtained during the survey are expressed as median price ratios (MPRs), or the ratio of a medicine’s median unit price across outlets to the median unit price in the Management Sciences for Health 2009 Price Indicator Guide [19], i.e. the international reference price (IRP). MSH international reference prices were selected as the most useful standard since they are updated frequently, always available and relatively stable. These prices are recent procurement prices offered by both not-for-profit and for-profit suppliers to developing countries for multi-source products. When no supplier prices are available, buyer prices are used. The MPRs will not be calculated until at least four procurement prices, or at least four public or private sector patient prices, are entered for the medicine in question. Generally, an MPR of 1 or less indicates an efficient public sector procurement system. Unlike procurement prices, there are no easy rules of thumb for determining if the MPRs for patient prices are high, low or about right. For the purposes of this discussion we use the following cut-off points of MPRs for patient prices to represent acceptable local price ratios: Patient prices in the public sector: MPR≤1.5; Patient prices in private pharmacies: MPR≤2.5 [20]. A Kruskal-Wallis test was applied, and p<0.05 was used to indicate significant difference.

According to the standard WHO/HAI methodology [18], the affordability of treating 22 common conditions was assessed by comparing the total cost of medicines prescribed at a standard dose with the daily wage of the lowest paid unskilled government worker, which was RMB 25.3333/day (USD 3.7152) at the time of the survey based on figures from the Department of Human Resources and Social Security of Shaanxi Province [21].

## Results

Of the outlets sampled, public procurement prices were available for 15 OBs and 28 generics out of the 47 medicines surveyed, public patient prices were collected from 50 public medicine outlets, and private patient prices were collected from 36 private pharmacies.

### Medicine Availability

The mean availability of OBs and LPGs was 8.9% and 26.5%, respectively, in the public sector, and 18.1% and 43.6% in the private sector. Analysis of survey medicines listed on the NEML [22] also found low availability. Separate availability analysis for 33 medicines listed on the NEML showed that mean availability in the public sector was 5.8% for OBs and 30.2% for LPGs, while mean availability in private sector retail pharmacies was 9.9% for OBs and 48.1% for LPGs.

Table 1 lists the availability of individual medicines in both the public and private sectors. Only 16 OBs were found in the public sector and 23 in the private sector. Only 10 LPGs in the public sector and 19 in the private sector had >50% availability.

**Table 1:** Availability of medicines in the public sectors and the private retail pharmacy sectors.

Availability	Public sector		Private sector	
	Originator brand	Lowest price generic	Originator brand	Lowest price generic
Medicines not found in any outlets	Aciclovir, aminophyline, amitriptyline, amoxicillin, atenolol, captopril, carbamazepine, cefradine, cephalexin, cimetidine ciprofloxacin, co-trimoxazole, diazepam, diclofenac, digoxin enalapril, erythromycin, fluconazole, glibenclamide, hydrochlorothiazide, ibuprofen, lisinopril, lovastatin, metronidazole, nifedipine retard, ofloxacin, paracetamol, phenytoin, ranitidine, rifampicin, sodium valproate	Aciclovir, atenolol, beclometasone inhaler, ciprofloxacin, glibenclamide, ibuprofen, ketoconazole, ofloxacin	Aciclovir, aminophylline, amitriptyline, atenolol, captopril, carbamazepine, cefradine, cephalexin, cimetidine, ciprofloxacin, co-trimoxazole, diazepam, diclofenac,enalapril, erythromycin, glibenclamide, hydrochlorothiazide, ibuprofen, lovastatin, metronidazole, ofloxacin, paracetamol, phenytoin, ranitidine, rifampicin	Atenolol, beclometasone inhaler, diazepam, ketoconazole
Medicines found in less than 25% of outlets	Azithromycin, beclometasone inhaler, ceftriaxone injection, fluoxetine, loratadine, losartan, metformin, omeprazole, simvastatin	Albendazole, amitriptyline, amlodipine, amoxicillin, atorvastatin, cefradine, cephalexin, diazepam, diclofenac, erythromycin, fluconazole, fluoxetine,lisinopril, loratadine, lovastatin,metformin, miconazole nitrate, paracetamol, salbutamol inhaler, simvastatin	Amoxicillin, azithromycin, beclometasone inhaler, ceftriaxone injection, digoxin, fluconazole fluoxetine, lisinopril, nifedipine retard, sodium valproate	Albendazole, amitriptyline, torvastatin, cefradine, ciprofloxacin, erythromycin, fluconazole, fluoxetine, glibenclamide, ibuprofen, lisinopril, losartan, miconazole nitrate, ofloxacin, paracetamol,
Medicines found in 25 to 50% of outlets	Albendazole, amlodipine, atorvastatin, gliclazide, ketoconazole, salbutamol inhaler	Carbamazepine, cimetidine, gliclazide, nifedipine retard, phenytoin, ranitidine, rifampicin, sodium valproate	Losartan, metformin, omeprazole, salbutamol inhaler, simvastatin	Aciclovir, amlodipine, cephalexin, diclofenac, loratadine, lovastatin metformin, phenytoin, simvastatin
Medicines found in 50 to 75% of outlets	NONE	Azithromycin, co-trimoxazole, digoxin,enalapril, hydrochlorothiazide	Amlodipine, atorvastatin, gliclazide, ketoconazole	Aminophylline, carbamazepine, ceftriaxone injection, digoxin, gliclazide, nifedipine retard, salbutamol inhaler, sodium valproate
Medicines found in over 75% of outlets	Miconazole nitrate	Aminophylline, captopril, ceftriaxone injection, metronidazole, omeprazole	Albendazole, loratadine, miconazole nitrate	Amoxicillin, azithromycin, captopril, cimetidine, co-trimoxazole, enalapril, hydrochlorothiazide, metronidazole, omeprazole, ranitidine, rifampicin

In all six regions, the mean availability of sampled medicines was higher in the private sector than the public sector, while generic medicines were more available than originator brands in both sectors. Analysis of medicine availability in primary health care institutions showed that mean availability of generic medicines ranged from 14.4% in Shangluo to 27.1% in Baoji. For originator brands, Yulin and Xi'an had the highest public sector availability of 7.1%, while Shangluo had the lowest public sector availability of 1.1%.

### Medicine Prices

Overall the public sector procures 28 generics at 0.75 times their IRPs, and 15 OBs at 8.49 times their IRPs. The MPRs of seven OBs – amlodipine (25.61), ceftriaxone injection (17.41), fluoxetine (108.39), gliclazide (11.43), loratadine (27.25), metformin (18.17), and omeprazole (56.59) – were more than ten times their IRPs. Fifteen LPGs were procured at lower prices than the IRPs; however, four medicines were more than five times the reference price, including amlodipine (5.16), diclofenac (21.87), enalapril (7.83) and loratadine (5.68).

Table 2 compares the price of medicines procured and then sold to patients in the public sector. Results show that final patient prices are 19.6% and 30.4% higher than procurement prices for 15 OBs and 28 generic equivalents, respectively. There were significant variations in mark-up rates (range: 14.7–48.1%; see Table S3) for 15 OBs, with 48.1% for simvastatin being the highest. Meanwhile, there were large differences in mark-up rates (range: 3.3–80.0%) among the 28 generic medicines.

**Table 2:** Median MPRs for OBs and LPGs in the public and private sectors.

	Public sector				Private sector	
Product type	No. ofmedicines	Median MPRsProcurement price	Median MPRsRetail price	% difference patientprices to procurement	No. ofmedicines	Median MPRsRetail price
Lowest price generic						
All	28	0.75	0.97	30.4%	37	1.53
Core list	18	1.59	1.84		20	1.46
Supplementary list	10	0.54	0.76		11	1.08
NEML	21	0.55	0.76		26	0.86
Originator brand	15	8.49	10.16	19.6%	15	8.36

OBs: Originator brands; LPGs: Lowest-priced generics; NEML: National Essential Medicine List (2009).

To reveal the relations between drug prices in the public sector and procurement prices, we analyzed the mark-up rates. For generic medicines procured at below IRP, the median mark-up was 27.2%, as shown in Table S3 (the highest mark-up was 80.0% for aminophylline). For generic medicines procured above IRP (such as diclofenac, enalapril and loratadine), the median mark-up was 15.1%. However, the actual add-on costs of medicines procured below IRP were less than those for generic medicines procured at higher prices than the IRPs (median add-on cost: 0.11 vs. 0.66). Therefore, high procurement prices are a major contributor to high retail prices (the higher the procurement price, the more profit a hospital can make through its mark-up).

In the private sector retail pharmacies, the median MPR for 15 OBs was 8.36 times the IRP, while the median MPR for 37 LPGs was 1.53 times the IRP (Table 2). Further analysis of 11 medicines for which both OBs and generically equivalent products were found showed that in the private sector, OBs cost 390.7% more, on average, than their generic equivalents. Thus, patients pay substantially more for originator brand medicines when lower-cost generics are unavailable.

In Table 3, only those medicines found in both public and private sector medicine outlets were included in the analysis to enable price comparison between the two sectors. Results showed that final patient price in the private sector is 16.5% lower than in the public sector for OBs, and 17.3% higher for generic equivalents.

**Table 3:** Median MPRs for medicines found in both the public and private sectors.

Product type	Median MPR Public sector patient prices	Median MPR Private sector patient prices	% difference private to public
Obs (n = 14 medicines)	9.98	8.83	−16.5%
LPGs (n = 27 medicines)	0.83	0.98	17.3%

OBs: Originator brands; LPGs: Lowest-priced generics.

**Figure 1:**
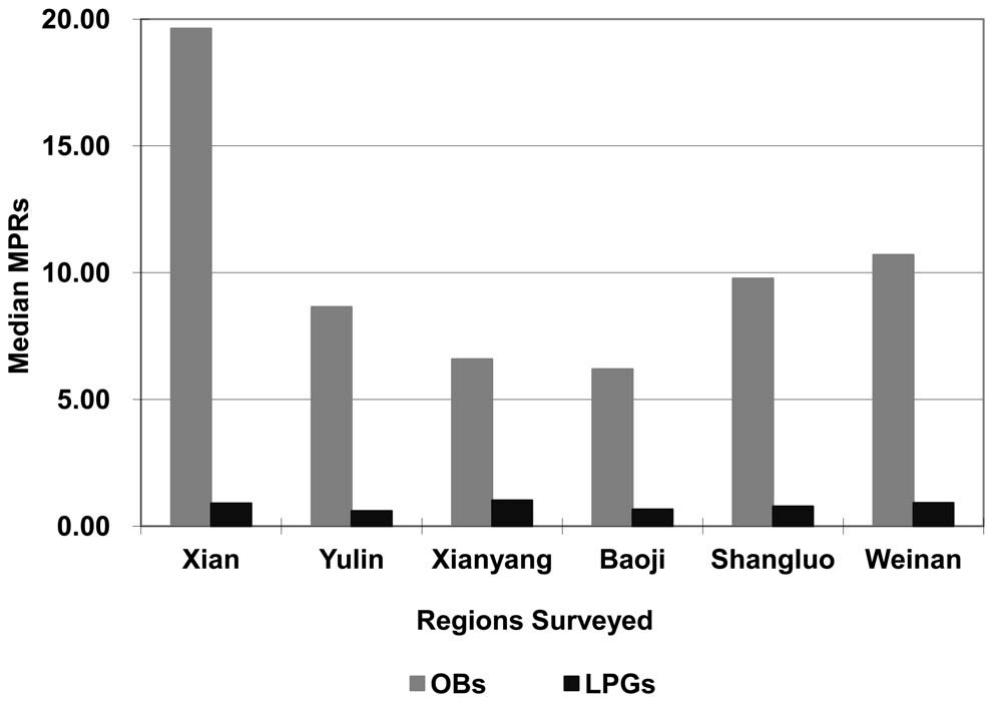
Regional Variations in Median MPRs in the Public Sectors. High and less variation in the median MPRs for OBs and LPGs, respectively, were observed in the public sectors across the six survey regions.

**Figure 2:**
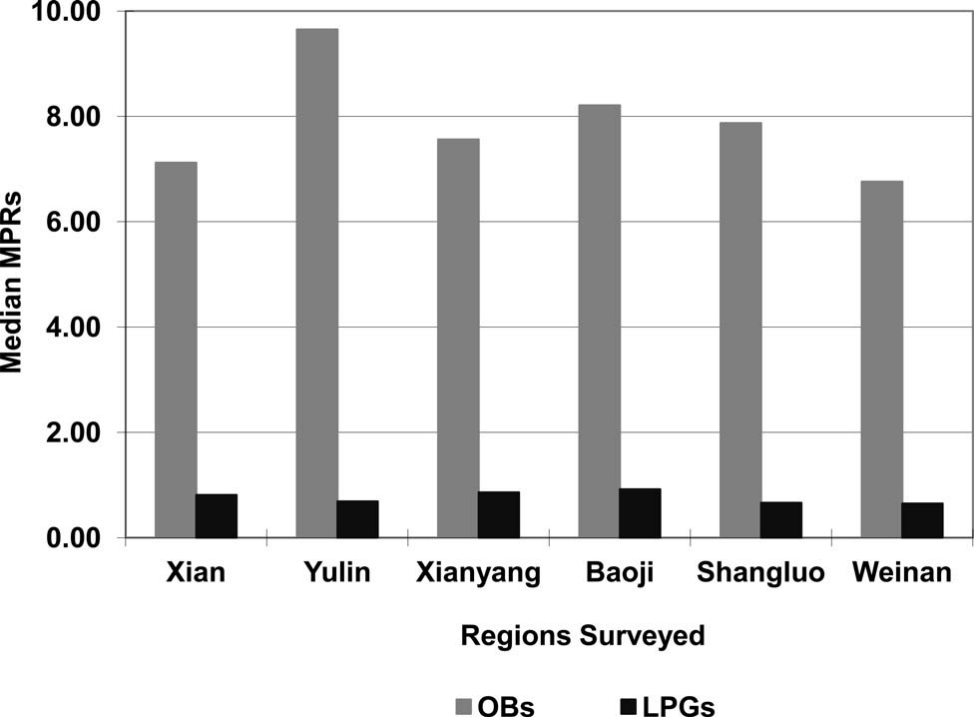
Regional Variations in Median MPRs in the Private Retail Sector Pharmacies. Less variations in the median MPRs for OBs and LPGs were observed in the private sectors across the six survey regions.

High variation across the six survey regions was noted for originator brands in the public sector. The highest median MPR, 19.63, was found in Xi'an whilst the lowest, 6.19, was in Baoji. Prices for lowest priced generics showed less variation across the regions with the lowest median MPR occurring in Yulin (0.6) and the highest in Xi'anyang (1.02) (Figure 1). The median MPRs for originator brands and generics in the private sector did not differ significantly across the six regions surveyed, and ranged from 6.76 in Weinan to 9.65 in Yulin for originator brands, and from 0.65 in Weinan to 0.92 in Baoji, respectively (Figure 2). However, because of the small sample size in each region (5 medicine outlets per sector, with each medicine available in at least 4), the results should be interpreted with caution.

Of all 94 medicines studied (47 OBs and 47 LPGs), eight were found in all six regions. A Kruskal-Wallis test revealed no significant difference in MPRs of these eight medicines across the six regions, with X^2^ (5, n = 48) = 0.888, p = 0.971.

### Affordability

The affordability of treatments for 11 different health conditions is listed in Table 4. In general, OB products were less affordable than the LPG equivalents in both the public and private sectors. The affordability of LPGs in the public sector was reasonable (with standard treatment costing a day's wage or less) for most conditions, except for amlodipine (1.7 days' wages), nifedipine retard (1.5 days' wages), simvastatin (2.2 days' wages) and diclofenac (2.3 days' wages).

**Table 4:** Number of days' wages of the lowest paid government worker needed to purchase standard treatments.

					Day’s wages to pay for treatment			
					public sector		private sector	
Condition	Drug name	Strength	No. ofunits a day	Duration days	OBs	LPGs	OBs	LPGs
Asthma	salbutamol	0.1 mg/dose	200	as needed	1.3	0.2	1.2	0.2
	beclometas	0.05 mg/dose	200	as needed	1.9	–	1.8	–
Diabetes	metformin	500 mg	3	30	5.7	–	4.9	0.3
	gliclazide	80 mg	1	30	1.6	0.6	1.3	0.5
Hypertension	amlodipine	5 mg	1	30	7.0	1.7	6.3	1.3
	captopril	l 25 mg	2	30	–	0.1	–	0.1
	lisinopril	10 mg	2	30	–	–	–	5.0
	losartan	50 mg	1	30	8.6		7.5	6.6
	nifedipine retard	20 mg	2	30	–	1.5	10.0	1.4
Hypercholesterolaemia	simvastatin	20 mg	1	30	5.4	2.2	4.5	1.9
	atorvastatin	20 mg	1	30	12.5		11.0	–
Depression	amitriptyline	25 mg	3	30	–	0.6		0.7
	fluoxetine	20 mg	1	30	12.3		11.5	2.9
Adult	ciprofloxacin	500 mg	2	7	–		–	0.1
respiratory	amoxicillin	500 mg	3	7	–	0.7	–	0.4
Infection	ceftriaxone	1 g/vial	1	as needed	3.7	0.1	–	0.1
Paediatric respiratory Infecion	co-trimoxazole	(80+400)mg/ml	2	7	–	0.0	–	0.0
Arthritis	diclofenac	50 mg	2	30	–	2.3	–	2.1
Ulcer	omeprazole	20 mg	1	30	16.9	0.6	15.6	0.8
	ranitidine	150 mg	2	30	–	0.2	–	0.2
Epilepsy	carbamazepine	100 mg	2	30	–	0.1	–	0.1
Viral infection	aciclovir	200 mg	5	5	–	–	–	0.4

OBs: Originator brands; LPGs: Lowest-priced generics.

In the private sector, LPGs had reasonable affordability for most conditions, while the following seven LPGs cost over a day’s wage: simvastatin, amlodipine, fluoxetine, lisinopril, losartan, nifedipine retard and diclofenac. The most affordable standard treatments were generally those for treating acute conditions like adult respiratory infection (0.1–0.4 days' wages). When OBs are prescribed and dispensed in the private sector, some treatment costs are surprisingly high. For example, treating peptic ulcer with omeprazole required 15.6 days' wages, while treating depression with fluoxetine cost 11.5 days' wages.

### Comprehensive Analysis of LPG Availability and Price

**Figure 3:**
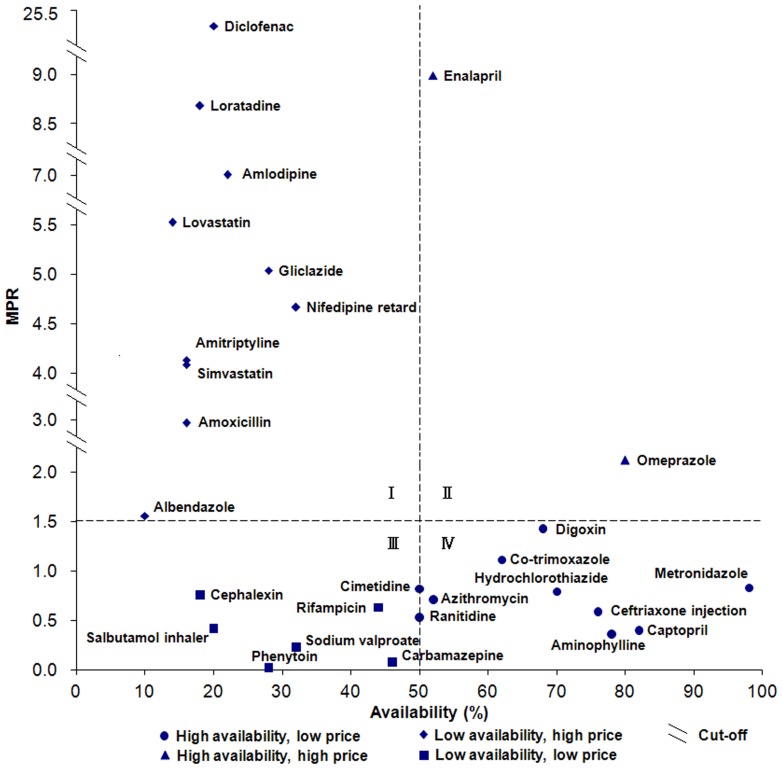
Comprehensive analysis of medicine availability and retail price in the public sector (LPGs). The availability score for each drug is depicted on the x-axis, while the y-axis shows the value of MPR.

**Figure 4:**
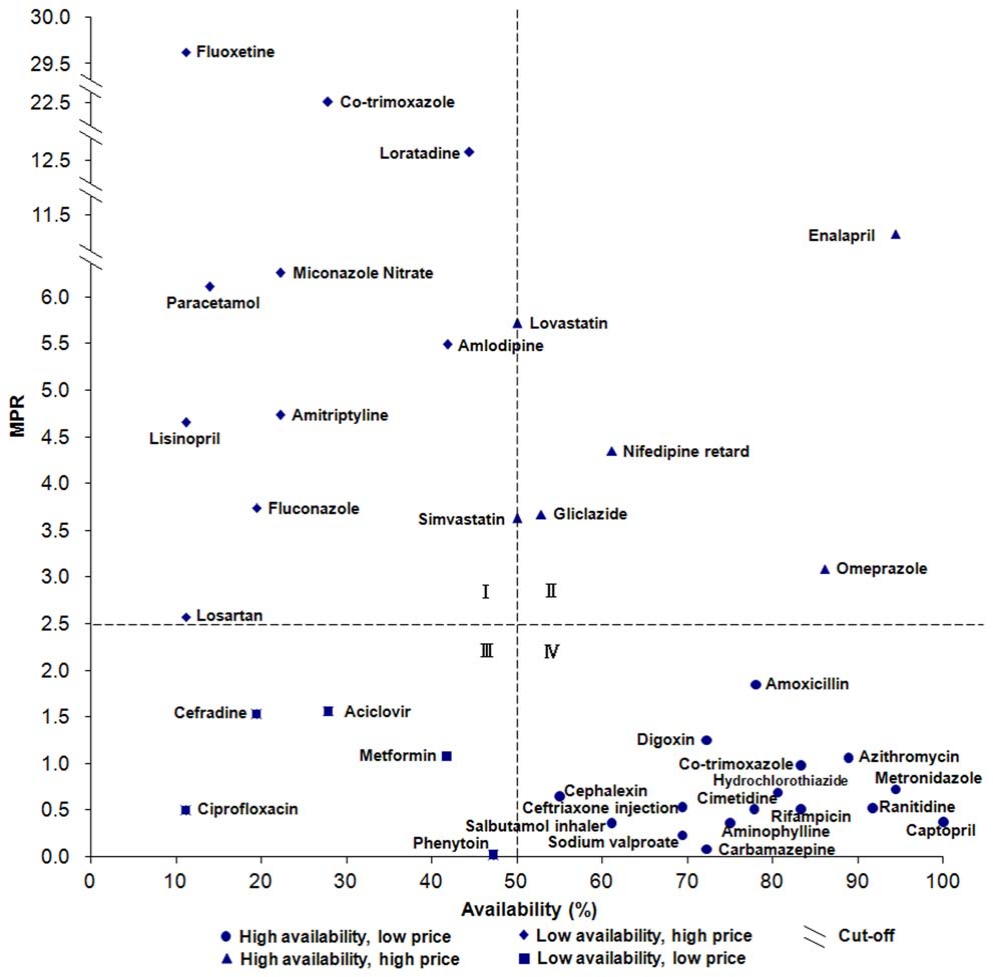
Comprehensive analysis of medicine availability and retail price in the private sector (LPGs). The availability score for each drug is depicted on the x-axis, while the y-axis shows the value of MPR.

Figure 3 displayed the availability and price of LPGs in the public sector. The availability score for each drug is depicted on the x-axis, while the y-axis shows the value of MPR. The figure can be divided into roughly four quadrants. The lower right quadrant (quadrant IV) contains drugs with low MPR and high availability, for example metronidazole which has 98.0% availability and a MPR of 0.83. The upper left quadrant (quadrant I) contains drugs with high MPR and low availability. In the case of these medicines, patients face both high costs and high difficulty in obtaining them. For example, diclofenac was available in only 20.0% of surveyed public sector hospitals and cost over 25 times the IRP. As shown in Figure 4, a slightly more optimistic situation exists in the private sector where 16 medicines are in quadrant IV, with good access and low cost, and ten medicines are in quadrant I, with low availability and high price. Two medicines for depression (amitriptyline and fluoxetine) are in quadrant I in both figures (fluoxetine was absent in Figure 3 owing to the lack of associated MPR data), making depression treatment potentially challenging in China.

Notably, some generics are easily accessed at low cost in both the public and private sectors, such as aminophylline, azithromycin, captopril, ceftriaxone injection, cimetidine, co-trimoxazole, digoxin, hydrochlorothiazide, metronidazole and ranitidine.

## Discussion

To date, four surveys [10]–[12], [23] have been carried out in China using the standardized, reliable methodology developed by WHO/HAI [18]. As the first study to apply the 2008 edition of the methodology to the less developed western region of China, the results of this medicine survey, together with three previously conducted surveys in eastern and central China, provide clearer insights into prices, availability, and affordability of medicines in China.

In 2009, the Chinese government launched a new round of healthcare reform, the main drug-related component of which is the implementation of a National Essential Medicines List. Monitoring of implementation and evaluation of the impact of essential drug policy in China is urgently needed. This study will fill in data gaps in the assessment of the prices, availability and affordability of essential medicines in China's western regions.

Results indicate that overall the government procurement agency is purchasing originator brands efficiently, but pays very high prices. These medicines are then on-sold to patients at over 19.6% more than the purchase prices as a result of add-on costs in the public sector distribution chain. Similar results were found in the Shandong [10], Shanghai [11] and Hubei [12] studies, and the MPRs of procurement prices were 0.62, 1.53 and 0.74 times the international reference prices for LPGs, while for OBs they were 6.30, 6.7 and 9.78, respectively. In Malaysia, the public procurement prices of medicines were moderately high relative to international prices, where the median MPRs of 2.44 and 1.09 were recorded for OBs and LPGs, respectively [24]. In contrast, seven Indian states [25]–[32] showed more efficient procurement and pricing in the public sector, where the lowest median MPR of LPGs was found in Haryana (0.33), and the highest in Rajasthan (0.96).

Lower availability of OBs than generic alternatives was seen in both the public and the private sectors, which could be attributed to their substantially higher prices. Medicines listed on the NEML had worse availability than other medicines in both sectors, especially in the public sector. Because of low availability of essential medicines in public hospitals, some patients had to purchase their medicines from retail pharmacies after consultation at a public health facility. No versions of diazepam and atenolol were found in any of the retail pharmacies surveyed (not even at other strengths). Unavailability of diazepam at retail pharmacies is probably because of its status in China as a strictly controlled psychotropic drug, which means that many pharmacies do not stock it. Atenolol was not found in any of the retail pharmacies, probably due to it not being used as a first line antihypertensive drug in China.

At the time of the survey, the zero mark-up policy had been conducted in all primary health care institutions in Yulin and Baoji. Although still very low, in the grass-roots hospitals surveyed, the highest mean availability of LPGs was in Baoji while that of OBs was in Yulin, and the prices were relatively low. The preliminary results showed that medicine availability was higher in two regions that had implemented a zero mark-up policy on drug sales than in regions without implementing such a policy. Further impacts of policy changes should be measured by establishing a monitoring system to regularly monitor medicine prices and availability [33]. To more fully study the medicine situation, the list of survey medicines should include more from both the NEML (2009) and the PEML (2010).

Although it is difficult to assess true affordability, treatments costing one day's wage or less (for a full course of treatment for an acute condition, or a 30-day supply of medicine for chronic diseases) are generally considered affordable. Notably, treatment costs refer to medicines only and exclude the additional costs of consultation and diagnostic tests. Further, since many people in China earn less than the lowest government wage, even treatments that appear affordable are too costly for the poorest segments of the population. Given that 16.6% of the population are living below the international poverty line, defined as income of less than $1/day, even treatments which appear affordable are out-of-reach for a substantial number of people. Meanwhile, even where individual treatments appear affordable, individuals or families who need multiple medications may quickly face unmanageable drug costs. An example is provided below of a family where the father has an ulcer and the child has asthma, treated with omeprazole and salbutamol inhaler, respectively. If the family income is equivalent to the lowest-paid government worker's salary, medicine costs assuming purchase of LPGs are 0.8 days' wages if purchasing via the public sector and 1.0 days' wages for the private sector. If OBs are purchased, treatment costs are 18.2 days' wages for the public sector and 16.8 for the private sector.

### Limitations of this Study

The present study has three limitations that need to be acknowledged and addressed. The first limitation concerns the availability of medicines. Availability data were collected at a specific point in time and involved a specific product dosage form and strength; as a result, the data may not reflect average availability over time. In addition, the availability is determined for the specific list of survey medicines, and does not account for the availability of alternate strengths or dosage forms, or therapeutic alternatives.

The second limitation involves the reliability of median price ratios, which may be skewed when international reference prices are based on limited data. In cases where very few supplier prices are available, or where there is no supplier price and the buyer price is used as a proxy, MPR results can be skewed by a particularly high or low international reference price [34].

The final limitation is that calculating affordability based on government worker wages may lead to over-optimistic results since a significant proportion of the population earns less than this.

### Conclusions

In Shaanxi Province, low availability was observed for all medicines surveyed, particularly for medicines listed on the NEML in the public and private sectors. Considerable price differences were seen between originator brands and generics in both sectors. OBs were more expensive than LPGs in both the public and private sectors. Medicines are often unaffordable for ordinary citizens. The price, availability and affordability of medicines in China should be improved to ensure equitable access to basic medical treatments, especially for the poor.

## Supporting Information

Table S1 Sample of public and private medicine outlets.
(DOCX)

Table S2 Medicines in the core and supplementary lists, and core essential medicines not surveyed.
(DOCX)

Table S3 Comparison of mark-ups between procurement and patient prices for OBs and LPGs in the public sector.
(DOCX)
