# Supplementary material for: Measuring Access to Medicines: A Survey of Prices, Availability and Affordability in Shaanxi Province of China
Source: PLoS One. 2013 Aug 1;8(8):e70836. doi: 10.1371/journal.pone.0070836 (PMC3731290; doi:10.1371/journal.pone.0070836)
Supplement: Table S1 — Sample of public and private medicine outlets. (DOCX) [file pone.0070836.s001.docx]

**Table S1 Sample of public and private medicine outlets**

| Area | Public sector | | |  | Private sector |
| --- | --- | --- | --- | --- | --- |
|  | Tertiary  hospital | Secondary  hospitals | Primary health care institutions |  | (pharmacies nearest to the  selected public hospitals) |
| Xian | 1 | 2 | 2 |  | 5 |
| Yulin | 1 | 2 | 2 |  | 5 |
| Xianyang | 1 | 2 | 2 |  | 5 |
| Baoji | 1 | 2 | 2 |  | 5 |
| Shangluo | 1 | 2 | 2 |  | 5 |
| Weinan | 1 | 2 | 2 |  | 5 |
| Total | 30 | | |  | 30 |
