# Supplementary material for: Measuring Access to Medicines: A Survey of Prices, Availability and Affordability in Shaanxi Province of China
Source: PLoS One. 2013 Aug 1;8(8):e70836. doi: 10.1371/journal.pone.0070836 (PMC3731290; doi:10.1371/journal.pone.0070836)
Supplement: Table S2 — Medicines in the core and supplementary lists, and core essential medicines not surveyed. (DOCX) [file pone.0070836.s002.docx]

**Table S2 Medicines in the core and supplementary lists, and core essential medicines not surveyed**

| No. | Medicine name | Strength | Dosage form | List |
| --- | --- | --- | --- | --- |
| 1 | salbutamol | 100 mcg/dose | inhaler | Global core |
| 2 | glibenclamide | 5 mg | cap/tab | Global core |
| 3 | atenolol | 50 mg | cap/tab | Global core |
| 4 | captopril | 25 mg | cap/tab | Global core |
| 5 | simvastatin | 20 mg | cap/tab | Global core |
| 6 | amitriptyline | 25 mg | cap/tab | Global core |
| 7 | ciprofloxacin | 500 mg | cap/tab | Global core |
| 8 | amoxicillin | 500 mg | cap/tab | Global core |
| 9 | ceftriaxone | 1 g/vial | injection | Global core |
| 10 | diazepam | 5 mg | cap/tab | Global core |
| 11 | diclofenac | 50 mg | cap/tab | Global core |
| 12 | omeprazole | 20 mg | cap/tab | Global core |
| 13 | albendazole | 200mg | cap/tab | Regional core |
| 14 | amlodipine | 5mg | cap/tab | Regional core |
| 15 | atorvastatin | 20mg | cap/tab | Regional core |
| 16 | beclometasone inhaler | 50mcg/dose | dose | Regional core |
| 17 | cephalexin | 250mg | cap/tab | Regional core |
| 18 | enalapril | 10mg | cap/tab | Regional core |
| 19 | fluoxetine | 20mg | cap/tab | Regional core |
| 20 | gliclazide | 80mg | cap/tab | Regional core |
| 21 | hydrochlorothiazide | 25mg | cap/tab | Regional core |
| 22 | ibuprofen | 400mg | cap/tab | Regional core |
| 23 | metformin | 500mg | cap/tab | Regional core |
| 24 | metronidazole | 200mg | cap/tab | Regional core |
| 25 | nifedipine retard | 20mg | cap/tab | Regional core |
| 26 | ranitidine | 150mg | cap/tab | Regional core |
| 27 | sodium valproate | 200mg | cap/tab | Regional core |
| 28 | co-trimoxazole | 80mg+400mg | cap/tab | Supplementary |
| 29 | paracetamol | 500mg | cap/tab | Supplementary |
| 30 | aciclovir | 200mg | cap/tab | Supplementary |
| 31 | carbamazepine | 100mg | cap/tab | Supplementary |
| 32 | cefradine | 500mg | injection | Supplementary |
| 33 | digoxin | 0.25mg | tab | Supplementary |
| 34 | fluconazole | 150mg | cap/tab | Supplementary |
| 35 | ketoconazole | 200mg | tab | Supplementary |
| 36 | losartan | 50mg | tab | Supplementary |
| 37 | phenytoin | 50mg | cap/tab | Supplementary |
| 38 | rifampicin | 150mg | tab | Supplementary |
| 39 | lovastatin | 20mg | cap/tab | Supplementary |
| 40 | ofloxacin | 200mg | tab | Supplementary |
| 41 | aminophyline | 100mg | tab | Supplementary |
| 42 | miconazole nitrate | 2% | cream | Supplementary |
| 43 | erythromycin | 250mg | tab | Supplementary |
| 44 | azithromycin | 250mg | cap/tab | Supplementary |
| 45 | cimetidine | 200mg | tab | Supplementary |
| 46 | lisinopril | 10mg | tab | Supplementary |
| 47 | loratadine | 10mg | tab | Supplementary |
