# Supplementary material for: Measuring Access to Medicines: A Survey of Prices, Availability and Affordability in Shaanxi Province of China
Source: PLoS One. 2013 Aug 1;8(8):e70836. doi: 10.1371/journal.pone.0070836 (PMC3731290; doi:10.1371/journal.pone.0070836)
Supplement: Table S3 — Comparison of mark-ups between procurement and patient prices for OBs and LPGs in the public sector. (DOCX) [file pone.0070836.s003.docx]

| Medicine | OBs | | | Medicine | LPGs | | |
| --- | --- | --- | --- | --- | --- | --- | --- |
|  | Procurement  price | Patient  price | Mark-up  Rate(%) |  | Procurement  price | Patient  price | Mark-up  Rate(%) |
| simvastatin | 6.86 | 10.16 | 48.1 | aminophylline | 0.20 | 0.36 | 80.0 |
| beclometasone inhaler | 2.55 | 2.93 | 17.1 | hydrochlorothiazide | 0.46 | 0.79 | 71.7 |
| ketoconazole | 5.57 | 6.45 | 15.8 | azithromycin | 0.43 | 0.71 | 65.1 |
| albendazole | 5.02 | 5.80 | 15.5 | cimetidine | 0.52 | 0.82 | 57.7 |
| salbutamol inhaler | 2.27 | 2.62 | 15.4 | ceftriaxone injection | 0.42 | 0.59 | 40.5 |
| miconazole nitrate | 8.49 | 9.80 | 15.4 | co-trimoxazole | 0.85 | 1.11 | 30.6 |
| amlodipine | 25.61 | 29.46 | 15.0 | salbutamol inhaler | 0.33 | 0.42 | 27.3 |
| gliclazide | 11.43 | 13.15 | 15.0 | metronidazole | 0.65 | 0.83 | 27.2 |
| fluoxetine | 108.39 | 124.67 | 15.0 | cephalexin | 0.65 | 0.76 | 16.9 |
| omeprazole | 56.59 | 65.06 | 15.0 | rifampicin | 0.55 | 0.63 | 14.5 |
| metformin | 18.17 | 20.89 | 15.0 | captopril | 0.35 | 0.40 | 14.3 |
| ceftriaxone injection | 17.41 | 20.01 | 14.9 | carbamazepine | 0.07 | 0.08 | 14.3 |
| atorvastatin | 2.22 | 2.55 | 14.9 | ranitidine | 0.47 | 0.53 | 12.8 |
| losartan | 2.90 | 3.33 | 14.8 | sodium valproate | 0.21 | 0.23 | 9.5 |
| loratadine | 27.25 | 31.25 | 14.7 | phenytoin | 0.02 | 0.02 | 0.0 |
|  |  |  |  | loratadine | 5.68 | 8.68 | 52.8 |
|  |  |  |  | amlodipine | 5.16 | 7.01 | 35.9 |
|  |  |  |  | simvastatin | 3.17 | 4.09 | 29.0 |
|  |  |  |  | omeprazole | 1.68 | 2.12 | 26.2 |
|  |  |  |  | diclofenac | 21.87 | 25.34 | 15.9 |
|  |  |  |  | lovastatin | 4.79 | 5.53 | 15.4 |
|  |  |  |  | amoxicillin | 2.58 | 2.97 | 15.1 |
|  |  |  |  | gliclazide | 4.38 | 5.04 | 15.1 |
|  |  |  |  | amitriptyline | 3.60 | 4.14 | 15.0 |
|  |  |  |  | enalapril | 7.83 | 8.99 | 14.8 |
|  |  |  |  | digoxin | 1.25 | 1.43 | 14.4 |
|  |  |  |  | nifedipine retard | 4.15 | 4.67 | 12.5 |
|  |  |  |  | albendazole | 1.51 | 1.56 | 3.3 |

**Table S3 Comparison of mark-ups between procurement and patient prices for OBs and LPGs in the public sector**

**OBs: Originator brands; LPGs: Lowest-priced generics**
